# Highly Sensitive Detection of Chymotrypsin Using Gold Nanoclusters with Peptide Sensors

**DOI:** 10.3390/mi17010107

**Published:** 2026-01-14

**Authors:** Siyuan Zhou, Cheng Liu, Haixia Shi, Li Gao

**Affiliations:** 1 Institute of Cancer Research, Affiliated Hospital of Jiangsu University, Zhenjiang 212013, China; 2 School of Life Sciences, Jiangsu University, Zhenjiang 212013, China; 3 P. E. Department, Jiangsu University, Zhenjiang 212013, China

**Keywords:** chymotrypsin, gold nanoclusters, limit of detection, high sensitivity

## Abstract

Pancreatic function tests are used to determine the presence of chronic pancreatitis, particularly in the early stage of the disease. Chymotrypsin is an indicator of pancreatic function and is thus related to pancreatic diseases. However, these methods often require specific equipment and cannot always meet on-site analysis requirements. Consequently, a highly sensitive detection method needs to be developed. This research employed graphene oxide modified with NHS sensors and peptides (RRHFFGC: Arginine-Arginine-Histidine-Phenylalanine-Phenylalanine-Glycine-Cysteine) tagged with gold nanoclusters (Au NCs) for the detection of chymotrypsin. The N-Hydroxysuccinimide-*(Polyethylene Glycol)*_4_-Dibenzocyclooctyne (NHS-PEG_4_-DBCO) and graphene oxide (GO)-N_3_ click reaction yielded GO-NHS material, appropriate for fluorescence quenching. The peptide chain was accurately broken with the introduction of chymotrypsin, and the Au NCs were situated far from the GO-NHS surface. The detection limit was 2.014 pg/mL. The results showed that the detection method had high sensitivity in comparison with the previous studies. This method is relevant to real samples due to its potential efficacy. Therefore, it is a promising method in the biomedical field.

## 1. Introduction

Chymotrypsin plays major roles in human diseases. Chymotrypsin influences various ailments, including pancreatic fibrosis, diabetes, indigestion, inflammation, hypertension, and several malignancies, notably cancer of the pancreas [1,2,3]. Chymotrypsin is an indicator of pancreatic function. Therefore, it is essential to develop a sensitive and reliable chymotrypsin detection technique for evaluating pancreatic function [4,5].

Currently, chymotrypsin can be identified using many methods, including high-performance liquid chromatography (HPLC) [6], mass spectrometry (MS) [7], electrochemical detection [8], colorimetric reaction detection [9], surface plasmon resonance (SPR) detection [10], paper-based flow sensor [11], and fluorescence resonance energy transfer (FRET) [12]. Chymotrypsin catalyzes the hydrolysis of peptide bonds of protein on the C-terminal side of tryptophan, tyrosine, phenylalanine, and leucine. A chymotrypsin-cleavable sequence with a cysteine terminus, RRHFFGC, was also reported [13]. However, these methods often require specific equipment which cannot always meet on-site analysis requirements [11]. Consequently, a highly sensitive detection method without specific equipment and meeting on-site analysis requirements should be developed in order that the disease can be found and treated at an early stage [14,15]. Nanomaterials provide chances to develop detection methods for chymotrypsin based on biosensors.

Gold nanoclusters (AuNCs), a type of nanomaterial, is made up of a few to hundreds of atoms of gold, have attracted significant interest due to their distinctive structures and photophysical characteristics [5,16,17]. Most AuNCs possess a diameter of 10 nm. Their dimensions can be likened to the electron’s Fermi wavelength, inhibiting the movement of valence electrons across the surface and resulting in notable electronic changes. This results in fascinating molecular attributes such as photoluminescence, HOMO–LUMO transitions, molecular chirality [18], size-dependent fluorescence, and distinct energy levels [19,20]. Moreover, Au NCs contrast with organic clusters due to their prolonged lifespans, superior biocompatibility, and substantial stokes shift, rendering them appropriate for chemical and biological sensing applications [18]. Owing to their colloidal stability, pronounced photoluminescence, and exceptional biocompatibility, Au NCs have been extensively utilized as fluorescence sensors for the detection of metal ions, various biomolecules, in vitro or in vivo imaging, and other study domains to date [21,22]. In the last two decades, Au NCs have attracted considerable attention as a fascinating category of luminescent materials. The structural analysis, primarily centered on size, was the predominant methodology utilized in the initial phases of Au NC research, facilitated by the rapid progress in organic chemistry. In summary, the rapid progress in the production techniques of luminous Au NCs has facilitated their application in biological and chemical sensing [23,24,25,26,27].

In this study, the peptide with the synthesized Au NCs resulted in a comprehensive peptide label that demonstrated fluorescence. The click reaction facilitated the attachment of a peptide to the surface of GO-N_3_ nanomaterials. GO-NHS was developed as a novel substance. The resultant reaction was characterized by its non-toxic nature, user-friendliness, and mildness. Chymotrypsin exhibited a remarkable sensitivity in detection when utilized in conjunction with the fluorescent peptide and GO-NHS. The proposed method utilized a peptide probe with a cysteine terminus and was promising with many advantages, such as rapid detection, simple operation, and low cost.

## 2. Materials and Methods

### 2.1. Chemical Reagents and Experimental Materials

Shanghai Sangon Co., Ltd. (Shanghai, China) synthesized the peptide sequence, RRHFFGC (Purity ≥ 98%). Shanghai Linc-Bio Science Co., Ltd. (Shanghai, China) was the supplier of chymotrypsin (Purity, 99%). Meiluo Technology Co., Ltd. was the source of NHS-PEG_4_-DBCO (Purity, 99%). Sigma-Aldrich (St. Louis, MO, USA) supplied the thrombin (Purity, 99%), lysozyme (Purity, 99%), bovine serum albumin (BSA, Purity ≥ 98%), IgG (Purity, 99%), HAuCl_4_, H_2_O, and 11-Mercaptoundecanoic acid (11-MUA, Purity, 99%). Zhejiang Tianhang Biotechnology Co., Ltd. (Huzhou, China) was the supplier of beef serum. From Sinopharm Chemical Reagent Co., Ltd. (Shanghai, China), additional reagents were procured. Shanghai Sangon Co., Ltd. was the supplier of the vascular endothelial growth factor (VEGF), Tubular dialyzer, and 0.45 μm filter. The BioTek Synergy H_4_ multipurpose microplate reader (Beijing Boao Biotechnology Co., Ltd., Beijing, China)was used to measure the fluorescence intensity. Origin 8.0 was used to process the data.

### 2.2. GO-NHS Synthesis

GO-N_3_ was bought from Jiangsu XFNANO Materials Tech Co., Ltd. (Nanjing, China) NHS-PEG_4_-DBCO reacted with GO-N_3_ nanomaterials by a click reaction with a ratio of 1:1 at room temperature. The reaction was carried out overnight. Then GO-NHS was developed as a novel substance.

### 2.3. AuNCs Synthesis

Ultrabright red-emitting fluorescent BSA-AuNCs were synthesized, as reported with minor modifications [28]. The HAuCl_4_ aqueous solution (5 mL, 10 mM) was combined with the BSA solution (5 mL, 50 mg/mL) under vigorous stirring. After 5 min, NaOH solution (0.5 mL, 1 mM) was added to the mixed solution continuously at 37 °C for 12 h under dark conditions. The prepared BSA-AuNCs were stored at 4 °C.

### 2.4. The Principle of the Experiment

**Figure 1:**
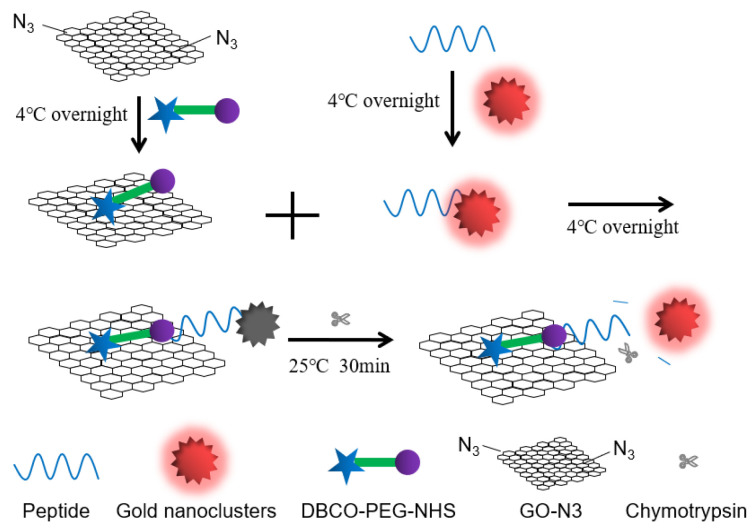
Scheme of detection of chymotrypsin by GO-NHS binding to peptide with Au NCs.

As shown in Figure 1, the peptide’s opposite amino group has the ability to react and attach to the GO-NHS surface. This extinguished the luminescence. The peptide was broken down by chymotrypsin, and the AuNCs were separated from the GO-NHS surface. Chymotrypsin was detected by comparing fluorescence intensity prior to and subsequent to its addition. The system was supplemented with peptide–AuNCs solution at a final concentration of 10 μg/mL, followed by the addition of a designated volume of GO-NHS solution. The mixing was conducted overnight at 4 °C, with a total volume of 400 μL. Following centrifugation at 12,000 rpm for 20 min, the supernatant was removed, and the volume was adjusted to 400 μL with ultrapure water. At this stage, fluorescence was quenched. Fluorescence intensity was then measured after adding a defined concentration of chymotrypsin and allowing the reaction to proceed for 30 min. The Au NCs were excited at a wavelength of 280 nm, and their emission was recorded from 580 nm to 700 nm in 2 nm increments. LOD was calculated based on Signal-to-Noise (LOD = 3S/N). LOD was calculated based on Signal-to-Noise (LOD = 3S/N), where S is the standard deviation of the blank signal and N is the slope of the calibration curve.

## 3. Results and Discussion

### 3.1. Characterization of Materials

**Figure 2:**
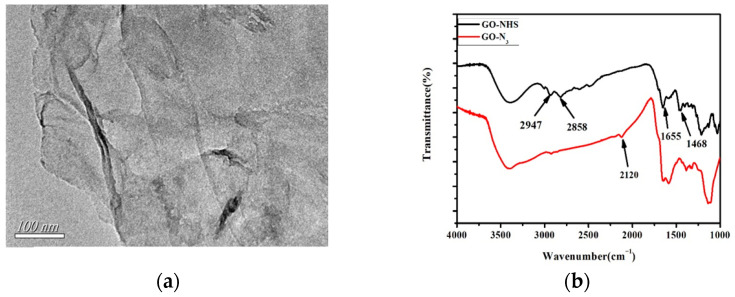
(**a**) Transmission Electron Microscope (TEM) of GO-N_3_; (**b**) GO-NHS solution and its FT-IR spectra. A peak at 2120 cm^−1^ was seen in the FT-IR spectra, which was ascribed to the absorption of N≡N.

**Figure 3:**
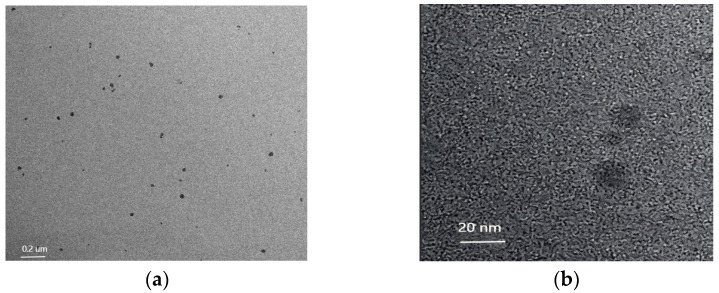
High-Resolution Transmission Electron Microscopy (HR TEM) for AuNCs. (**a**) Magnification was 8000 and beam energy was 200 kV. (**b**) Magnification was 60,000 and beam energy was 200 kV.

**Figure 4:**
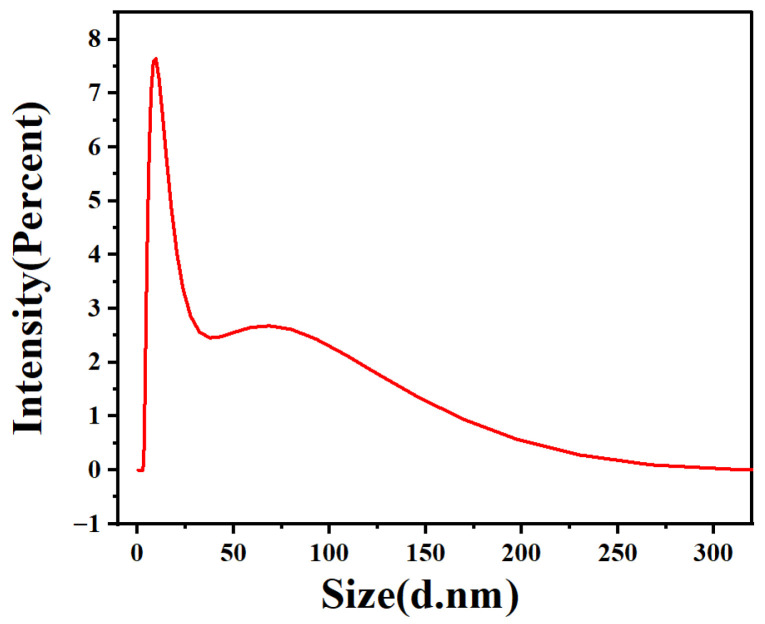
Dynamic Light Scattering (DLS) for AuNCs. Most AuNCs possessed a diameter of 10 nm.

A Transmission Electron Microscope (TEM) of GO-N_3_ is shown in Figure 2a. The typical GO morphology and inherent wrinkles and folds of GO could be observed. As shown in Figure 2b, following the azide group functionalization of GO, a peak at 2120 cm^−1^ was seen in the FT-IR spectra, which was ascribed to the absorption of N≡N. This demonstrated the effective acquisition of azide-functionalized GO. Accordingly, the absorption peaks of GO-NHS were 1466 cm^−1^ (C-H shear vibration), 1631 cm^−1^ (C=O stretching vibration), 2853 cm^−1^ (C-H symmetric stretching vibration), and 2924 cm^−1^ (C-H asymmetric stretching vibration) [29,30]. As shown in Figure 3, High-Resolution Transmission Electron Microscopy (HR TEM) for AuNCs was carried out. Figure 3a,b are shown with different magnifications (8000× and 60,000×). The diameters of most AuNCs were about 10 nm. Dynamic Light Scattering (DLS) for AuNCs is shown in Figure 4. This further confirmed the diameters of AuNCs.

**Figure 5:**
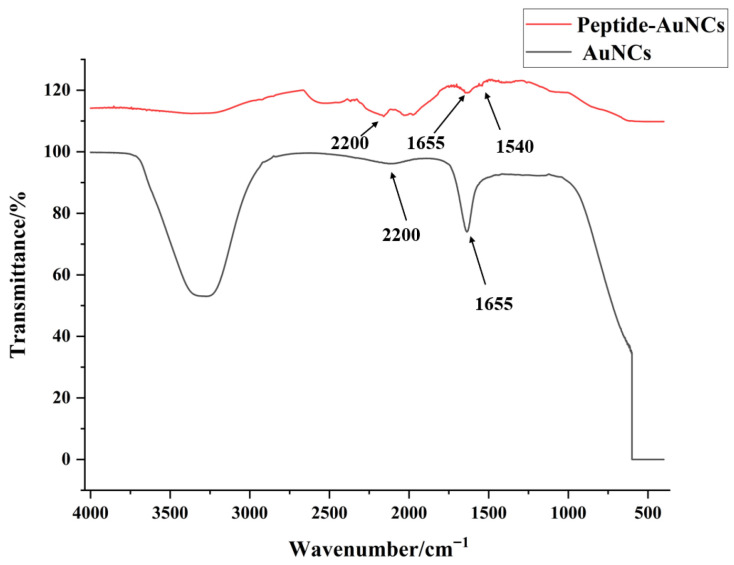
FT-IR spectra of Au NCs and peptide–Au NCs. Peptide-functionalized AuNCs showed two characteristic absorption bands at ~1655 cm^−1^ and ~1540 cm^−1^, corresponding to the amide I and amide II vibrations of the peptide backbone. At the same time, a distinct band around 2200 was observed in AuNCs.

FT-IR spectra of Au NCs and peptide–Au NCs are shown in Figure 5. Peptide-functionalized AuNCs showed two characteristic absorption bands at ~1655 cm^−1^ and ~1540 cm^−1^, corresponding to the amide I and amide II vibrations of the peptide backbone. At the same time, a distinct band around 2200 cm^−1^ was observed in AuNCs, which was significantly suppressed after peptide conjugation, indicating a strong interaction between the peptide ligands and the AuNC surface. These results confirmed the successful functionalization of AuNCs with peptides [31,32].

### 3.2. Optimization of P-Au NCs Concentration

**Figure 6:**
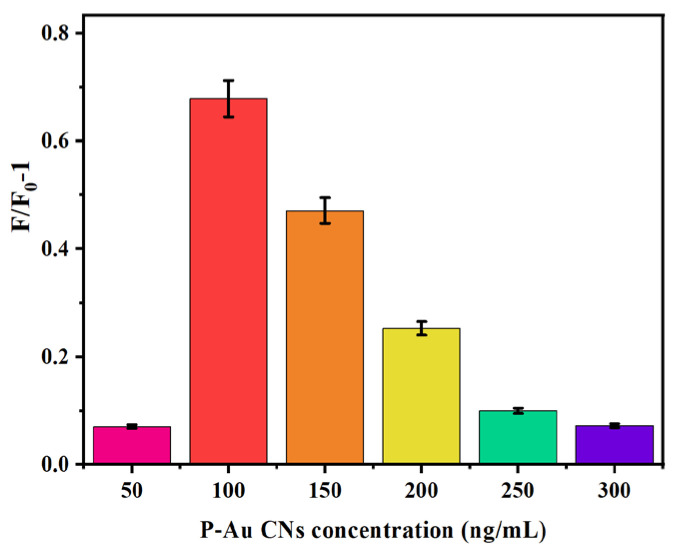
Variation in fluorescence intensity with different P–Au NCs concentrations. Various concentrations of P-AuNCs (50, 100, 150, 200, 250, and 300 ng/mL) were combined with GO-NHS (30 μg/mL), and 100 ng/mL of chymotrypsin was included into each combination.

Therefore, chymotrypsin can cut the peptide (RRHFFGC) coating around the AuNCs [13]. Various concentrations of P-AuNCs (50, 100, 150, 200, 250, and 300 ng/mL) were combined with GO-NHS (30 μg/mL), and the first fluorescence intensity (F_0_) was measured. Subsequently, 100 ng/mL of chymotrypsin was added to each combination, and the fluorescence intensity (F) was quantified during a 30 min incubation at ambient temperature. The relative alteration in fluorescence intensity, denoted as F/F_0_–1, was analyzed across various P-AuNC concentrations. The results are illustrated in Figure 6. The most significant alteration in fluorescence intensity occurred when 100 ng/mL P-AuNCs were exposed to chymotrypsin. While less than 100 ng/mL P–AuNCs could digest 100 ng/mL of chymotrypsin, more than this concentration improved the fluorescence intensity. Therefore, we found that the concentration of 100 ng/mL P-AuNCs was ideal for the experiment, due to the lower background. Therefore, this concentration was deemed ideal.

### 3.3. Optimization of GO-NHS Concentrations

**Figure 7:**
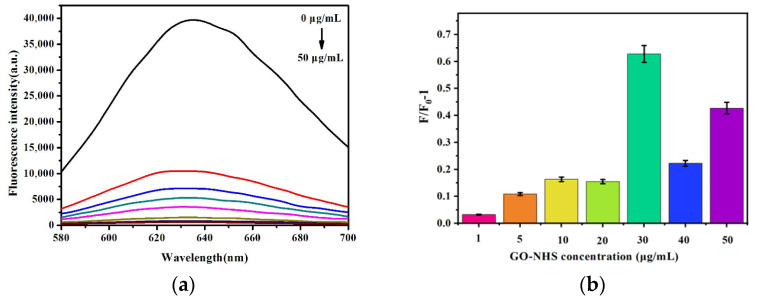
(**a**) The fluorescence intensity caused by different concentrations of GO-NHS (1, 5, 10, 20, 30, 40, and 50 μg/mL). Chymotrypsin (100 ng/mL) was then added. The resulting fluorescence intensity (F) was measured after a 30 min incubation at room temperature. (**b**) The relative fluorescence change (F/F_0_–1) was assessed for each concentration of GO-NHS.

GO-NHS solutions at varying concentrations (1, 5, 10, 20, 30, 40, and 50 μg/mL) were introduced into the system, and the initial fluorescence intensity (F_0_) is recorded in Figure 7a. Chymotrypsin (100 ng/mL) was then added, and the resulting fluorescence intensity (F) was measured after a 30 min incubation at room temperature. As illustrated in Figure 7b, the relative fluorescence change (F/F_0_–1) was assessed for each concentration of GO-NHS. Less than 30 μg/mL of GO-NHS was not enough for immobilizing P-AuNCs. More than 30 μg/mL of GO-NHS could quench the fluorescence of P-AuNCs when 100 ng/mL P-AuNCs were exposed to 100 ng/mL chymotrypsin. The most notable fluorescence alteration transpired at 30 μg/mL GO-NHS, which was consequently designated as the best concentration for ensuing tests.

### 3.4. Kinetic Analysis

**Figure 8:**
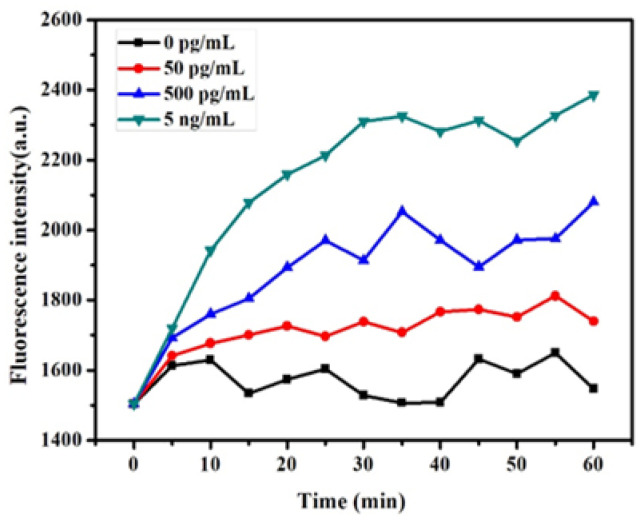
The fluorescence intensity of the sensor with three types of concentrations (50 pg/mL, 500 pg/mL, and 5 ng/mL) of chymotrypsin at different times, P-AuNCs (10 μg/mL), and GO-NHS (30 μg/mL).

The system was prepared by introducing P-AuNCs (100 ng/mL) and GO-NHS (30 μg/mL), followed by the addition of chymotrypsin at graded concentrations (50 pg/mL, 500 pg/mL, and 5 ng/mL). The resulting fluorescence variation, expressed as (F/F_0_–1), was monitored over time. As shown in Figure 8, fluorescence increased rapidly within the first 20 min and then stabilized around 30 min for all chymotrypsin concentrations. After chymotrypsin digested the peptide, the AuNCs were released. The resulting fluorescence intensity increased. However, once the chymotrypsin was used, the resulting fluorescence intensity became stabilized. Based on these observations, a reaction time of 30 min was chosen.

### 3.5. Sensitivity Detection

**Figure 9:**
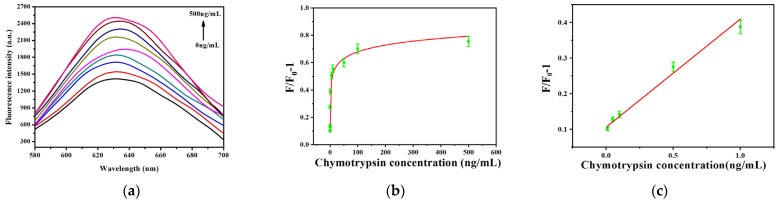
(**a**) Detection of different concentrations of chymotrypsin using Au NCs–peptide sensors. 100 ng/mL of P-AuNCs, 30 μg/mL of GO-NHS, and different concentrations of chymotrypsin (10 pg/mL, 50 pg/mL, 100 pg/mL, 500 pg/mL, 1 ng/mL, 5 ng/mL, 10 ng/mL, 50 ng/mL, 100 ng/mL, and 500 ng/mL) were added into the system, followed by a 30 min incubation; (**b**,**c**) LOD determined by 3S/N was 2.014 pg/mL.

The sensor’s sensitivity was assessed under optimized circumstances for P-AuNCs concentration, GO-NHS concentration, and reaction duration. Precisely, 100 ng/mL of P-AuNCs and 30 μg/mL of GO-NHS were incorporated into the system. The baseline fluorescence intensity (F_0_) was measured. A series of chymotrypsin concentrations (10 pg/mL, 50 pg/mL, 100 pg/mL, 500 pg/mL, 1 ng/mL, 5 ng/mL, 10 ng/mL, 50 ng/mL, 100 ng/mL, and 500 ng/mL) were introduced into the system, followed by a 30 min incubation. Fluorescence intensity (F) was recorded, and the results were presented in Figure 9a. Beer’s law assumes a strictly linear dependence of the absorbance from concentration. This linear correlation translates into a linear dependence of the absorbance for low concentrations. Usually, chemical interactions and instrumental imperfection were made responsible for experimental deviations from this linearity [33]. As shown in Figure 9b, the fluorescence intensity showed a consistent trend. According to the data, there was a significant linear association between chymotrypsin concentration and the change in fluorescence intensity from 0.01 ng/mL to 1 ng/mL in Figure 9c. The regression equation describing the linear relationship was y = 0.3033x + 0.1065, R^2^ = 0.98. The limit of detection (LOD) determined by 3S/N was 2.014 pg/mL. As reported by Piovarci et al. [34], the detection limit was 1.40 nM (35 ng/mL). Gupta et al. [35] developed a fluorescence biosensor with a detection limit of 0.130 nM (3.25 ng/mL). The results showed that the detection method had high sensitivity.

As shown in Table 1, the linear range and the LOD of this work and some of the other literature were compared. The LOD of this work had an obvious advantage compared with those of other works.

As shown in Table 1, Porous Coordination Network (PCN)@graphene oxide (GO) @ gold nanoparticle (AuNP) as a composite nanomaterial combined with peptide was used to detect chymotrypsin. The LOD was 3.91 pg/mL [4]. In this study, chymotrypsin was used to digest the peptide between GO and AuNCs. The LOD was 2.014 pg/mL, which was improved [13,34,35,36].

### 3.6. Selectivity Analysis

**Figure 10:**
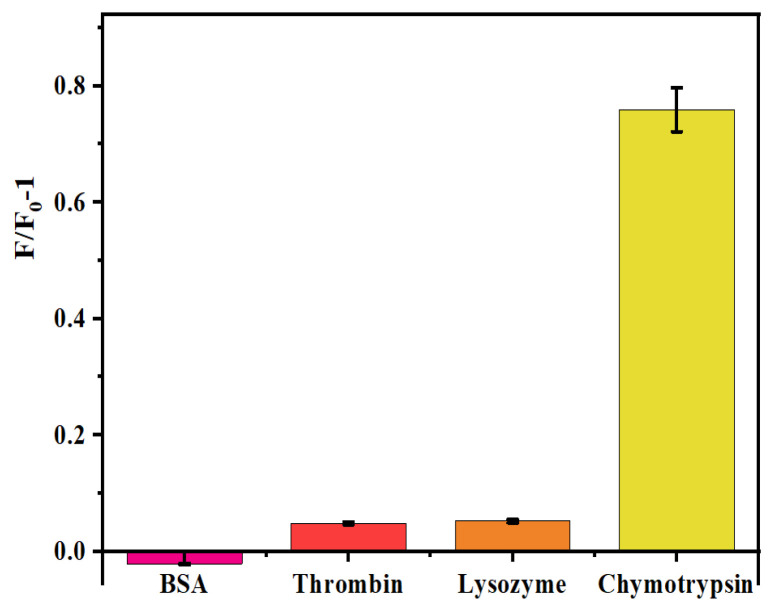
The fluorescence intensity caused by 100 ng/mL IgG, chymotrypsin, thrombin, BSA, VEGF, and lysozyme. A total of 100 ng/mL P-AuNCs, 30 μg/mL GO-NHS, and equivalent quantities (100 ng/mL) of chymotrypsin, IgG, VEGF, thrombin, BSA, and lysozyme were individually administered, followed by a 30 min reaction interval. The value of chymotrypsin to the value of lysozyme, thrombin, and BSA was very significant (*p* < 0.01) in this case.

A selectivity test was performed to illustrate the sensor’s specificity for chymotrypsin. A final dose of 100 ng/mL P-AuNCs and 30 μg/mL GO-NHS was introduced to the system, and the initial fluorescence intensity (F_0_) was measured. Subsequently, equivalent quantities (100 ng/mL) of chymotrypsin, IgG, VEGF, thrombin, BSA, and lysozyme were individually administered, followed by a 30 min reaction period. Subsequently, the fluorescence intensity (F) was quantified. Chymotrypsin can specifically digest the peptide. Other proteins could not react with this peptide. Figure 10 illustrates that chymotrypsin elicited a notable fluorescence alteration in contrast to the other chemicals, suggesting the sensor’s pronounced selectivity.

### 3.7. Application in Real-World Samples

To evaluate the sensor’s stability and practical applicability, chymotrypsin was tested in beef serum (Tianhang Biotechnology Co., Ltd., Huzhou, China) at three concentrations (10, 50, and 100 pg/mL) diluted in PBS buffer. The obtained information from chymotrypsin added in serum was detected using the biosensor. Each concentration was tested three times in normal serum. The results illustrated in Table 2 indicate recovery rates from 93.129% to 115.40%, accompanied by relative standard deviations of 1.374% to 3.280%, meeting the practical application requirements. These results demonstrate that the sensor is suitable for real-world detection.

## 4. Conclusions

This study used gold nanoclusters labeled with peptide and graphene oxide modified with NHS sensors to detect chymotrypsin. A click reaction led to the creation of GO-NHS material, which is suitable for quenching fluorescence. The fluorescent peptide and GO-NHS material can then mix, allowing the amino group at the opposite end of the peptide to covalently bind to the GO-NHS surface, resulting in quenching the fluorescence. The peptide was cleaved and the fluorescence returned when chymotrypsin was introduced. The sensor achieved a detection limit as low as 2.014 pg/mL with 30 min for detection time. The experimental conditions were mild, and the procedure was straightforward. NHS modification of the GO surface enhanced the sensor’s sensitivity, which was notably higher than that of conventional methods. This work offers a novel and effective approach for chymotrypsin detection.

## Figures and Tables

**Table 1:** A comparison of our method with other methods for chymotrypsin detection.

Methods	Analyst	Detection Time	Linear Range	LOD	References
Fluorescence detection	Grapheneoxide/nucleic acid-stabilized silver nanoclusters hybrid materials	20 min	0–50 ng/mL	3 ng/mL	[13]
Dynamic Light Scattering (DLS)	Gold nanoparticles (AuNPs)@ 6-mercapto-1-hexanol (MCH) @ β-casein	30 min	0.67 ± 0.05 nM	35 ng/mL	[34]
Fluorescence detection	Squaraine dye-based far-red fluorescent probe	60 min	0–0.5 nM	3.25 ng/mL	[35]
Fluorescence detection	Porous Coordination Network (PCN)	30 min	0.01–1 ng/mL	3.91 pg/mL	[4]
Fluorescence detection	Paper-based sensor	15 min	0–8 μg/mL	330 ng/mL	[36]
Fluorescence detection	Grapheneoxide@goldnanoclusters@peptide	30 min	0–1.0 ng/mL	2.014 pg/mL	This study

**Table 2:** Results for the determination of chymotrypsin in the serum (*n* = 3).

Samples	Added (pg/mL)	Detected (pg/mL)	Recovery (%)	RSD (%)
1	10	11.540	115.40	2.004
2	50	46.564	93.129	1.374
3	100	109.087	109.087	3.280

## Data Availability

The data supporting this study’s findings are available from the corresponding author upon reasonable request.
